# Comparative Analysis of Envelope and Triangular Flap Designs in Reducing Postoperative Sequelae Following Mandibular Third Molar Disimpaction

**DOI:** 10.7759/cureus.91573

**Published:** 2025-09-03

**Authors:** Mimansa Daftary, Nandakishore Donepudi, Deepak Thakur, Saubhagya S Agrawal, Nakul Chaudhary, Saayam Jain

**Affiliations:** 1 Department of Oral and Maxillofacial Surgery, Teerthanker Mahaveer Dental College and Research Centre, Moradabad, IND

**Keywords:** envelope, extraction, flap, mandibular third molar, surgical, triangular

## Abstract

Introduction: Surgical removal of impacted mandibular third molars often results in postoperative complications, such as pain, swelling, trismus, and periodontal changes, which can affect patient recovery. The choice of the flap design may influence these outcomes. This study compared the envelope flap and triangular flap with the modified Ward’s incision to minimize postoperative sequelae, focusing on pain, facial swelling, maximum mouth opening, and probing pocket depth, and to provide evidence-based insights for optimizing surgical outcomes in third molar disimpaction.

Materials and methods: A prospective comparative study was conducted at the Department of Oral and Maxillofacial Surgery, involving 40 systemically healthy patients (American Society of Anesthesiologists or ASA I or II) aged 18-50 years with mesioangular, distoangular, or vertically impacted mandibular third molars. Patients were assigned to two groups (n=20 each): Group 1 (triangular flap with modified Ward’s incision) and Group 2 (envelope flap). Standardized surgical protocols were followed, with local anesthesia and primary closure using 3-0 silk sutures. Postoperative outcomes such as pain (visual analog scale), maximum mouth opening (interincisal distance), facial swelling (metric facial measurements), and probing pocket depth were assessed on days 1, 3, and 7 and at 3 months. Data were analyzed using independent t-tests and repeated-measures analysis of variance with a significance threshold of p≤0.05.

Results: Both groups showed significant improvements in all parameters over time (p<0.001). The envelope flap group had less facial swelling (117.68±6.27 mm on day 1, further reduced by day 7) than the triangular flap group (119.80±5.29 mm on day 1, increased on days 3 and 7). Maximum mouth opening improved faster in the envelope group (21.50±4.47 mm on day 1, significant by day 3) than the triangular group (22.70±5.27 mm on day 1). Pain decreased similarly, from 7.90±0.85 (envelope) and 7.80±0.83 (triangular) to 1.25±0.91 and 1.80±0.83 by day 7, with no notable differences. The probing pocket depth increased temporarily (mean difference = 1.47 mm, p<0.001) but equalized by 3 months (p=0.157). The simpler design of the envelope flap likely reduced tissue trauma and inflammation, enhancing swelling and trismus outcomes.

Conclusion: The envelope flap outperformed the triangular flap in reducing postoperative swelling and enhancing early mouth opening recovery, making it preferable for routine mandibular third molar extractions. Both techniques ensured comparable pain control and periodontal healing, supporting their use based on case-specific requirements.

## Introduction

Tooth impaction, particularly of the third molar, has been a significant focus in dental surgery because of its prevalence and associated complications [1]. An impacted tooth fails to erupt into its proper position owing to malposition, lack of space, or other physical impediments [2]. The third molar, commonly known as the wisdom tooth, is the most frequently impacted tooth, affecting approximately 22% of individuals in Kerala [3] and 73% in Europe [1]. This high incidence underscores the necessity for surgical intervention, as untreated symptomatic third molars can lead to severe complications, including cyst formation, resorption of adjacent teeth, and periodontal issues [4].

Surgical extraction of the third molars is a complex procedure influenced by factors such as tooth location, angulation, and the presence of infections [4,5]. The procedure often results in postoperative sequelae that affect patients’ quality of life, including pain, swelling, trismus (jaw stiffness), fibrinolytic osteitis, and compromised periodontal health of the adjacent teeth [5]. The type of surgical flap used to access the impacted tooth plays a critical role in determining these outcomes. In third molar surgery, flap design influences postoperative complications, particularly the periodontal health of the adjacent second molar [6]. As posited by Mohajerani et al. [7], the reduction of surgical complications subsequent to third molar extraction is a significant concern and may be realized through the strategic design of an optimal flap.

Two commonly employed flap designs in third molar surgery are the envelope flap (Koener incision) and the triangular flap (modified Ward incision) [8]. The envelope flap involves a horizontal incision parallel to the gingival margin without vertical releasing incisions, offering a conservative approach with ease of repositioning and a reduced risk of wound dehiscence. In contrast, the modified Ward’s incision incorporates a vertical anterior incision starting from the distal aspect of the first molar, extending along the buccal crevice of the second molar, and continuing to the external oblique ridge [8,9]. This design provides superior visualization and access to the surgical site but may compromise the blood supply and extensibility compared to the envelope flap.

The primary aim of this prospective study was to assess the clinical outcomes of two distinct flap designs, the envelope flap and the triangular flap, in the context of postoperative sequelae following mandibular third molar disimpaction. This study compared the effectiveness of these surgical approaches in minimizing the complications that affect patient recovery and quality of life. Specifically, the objectives were to evaluate postoperative sequelae based on four key parameters: pain, trismus or jaw stiffness as assessed by maximum mouth opening, swelling (measured through facial dimensions), and probing pocket depth. By systematically analyzing these factors, this study aimed to provide evidence-based insights into the relative advantages and limitations of each flap design for third molar surgery.

## Materials and methods

This prospective, comparative study was conducted at the Department of Oral and Maxillofacial Surgery, Teerthanker Mahaveer Dental College and Research Centre, Moradabad, India, over a period of 18 months. Ethical clearance was obtained from the Ethical Committee of the Teerthanker Mahaveer University (TMDCRC/IEC/TH/22-23/OMFS 02). Informed written consent was obtained from all patients, adhering to the principles outlined in the Declaration of Helsinki.

Eligible patients for the study were individuals aged 18-50 years, of both sexes, presenting with mesioangular, distoangular, or vertically impacted mandibular third molars. These patients were required to be systemically healthy, specifically categorized as American Society of Anesthesiologists (ASA) Physical Status Classification I or II. ASA I indicates a healthy individual with no systemic disease, whereas ASA II includes patients with mild systemic disease (e.g., well-controlled hypertension or diabetes) that does not interfere with daily activities or surgical procedures under local anesthesia [10]. Patients also needed to express their willingness to undergo lower third molar disimpaction and commit to attending follow-up visits on postoperative days 1, 3, and 7 and at 3 months.

The exclusion criteria were established to ensure patients' safety and homogeneity. Patients with Position C impactions, as defined by the Pell and Gregory classification [11], were excluded. In this classification, Position C refers to the mandibular third molars, where the occlusal surface of the impacted tooth is at or below the cervical line of the adjacent second molar, indicating a deeply impacted tooth with increased surgical complexity. Pregnant or lactating women were excluded because of the potential risks associated with surgical stress and postoperative medications. Individuals with mental disabilities that could impair informed consent or compliance with the follow-up protocols were also excluded. Patients diagnosed with oral submucosal fibrosis, a chronic condition characterized by mucosal fibrosis and reduced mouth opening, were excluded to avoid confounding factors related to baseline trismus and compromised healing.

The sample size was calculated using the G*Power software (version 3.1.9.2; Heinrich Heine University, Düsseldorf, Germany). An effect size of 0.8 was derived from a previous study evaluating post-impaction trismus for estimation purposes [9]. The analysis determined that 20 patients per group (total sample size = 40) would provide 80% statistical power, with a significance level (alpha) of 0.05. This computation was based on a two-tailed independent t-test to ensure robust intergroup comparisons.

The non-randomized design was chosen to simplify patient allocation based on clinical presentation and the surgeon's preference, and to reflect real-world clinical practice, where flap selection is often determined by case-specific factors such as tooth angulation and accessibility. Patients were sequentially assigned to either Group 1 (triangular flap with modified Ward’s incision, n=20) or Group 2 (envelope flap, n=20) based on the surgeon’s assessment during the preoperative evaluation. 

The surgical protocol was standardized for both groups. A detailed clinical case history was recorded, supplemented by thorough clinical and radiological examinations using panoramic radiographs to assess tooth position and impaction status. All procedures were performed under local anesthesia using 2% lignocaine hydrochloride with epinephrine (1:80,000) (Xylocaine 2% with adrenaline, AstraZeneca, Cambridge, United Kingdom) administered via a 2 mL syringe (Dispovan, Hindustan Syringes & Medical Devices Ltd., Faridabad, India). The surgical site was prepared with 10% povidone-iodine solution (Betadine, Win-Medicare Pvt. Ltd., New Delhi, India) for antisepsis, and the patients were draped to maintain a sterile field.

**Figure 1:**
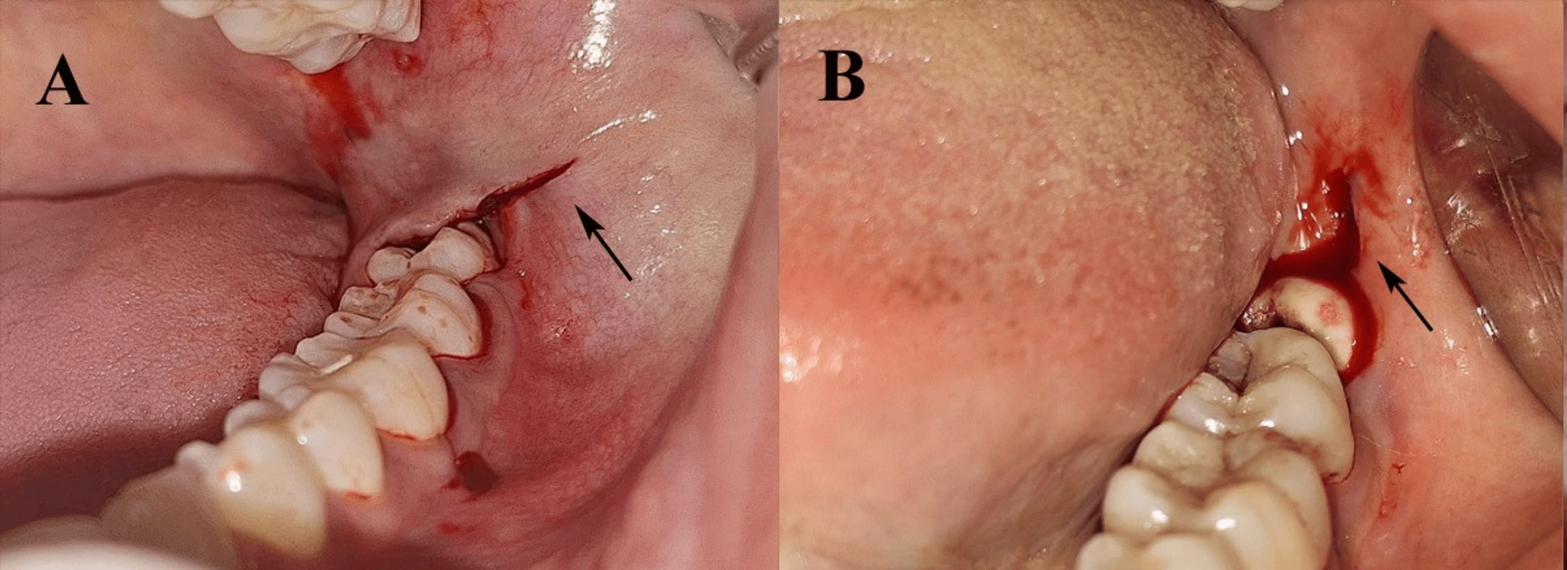
Types of incisions used for disimpaction of mandibular third molar: (A) triangular flap with modified Ward's incision and (B) envelope flap. Original intraoral images of patients from the study were used with the patients’ permission.

In Group 1, the triangular flap with the modified Ward’s incision was employed, starting with a vertical anterior incision from the distal aspect of the mandibular first molar, extending along the tooth, followed by a linear incision along the buccal gingival crevice of the second molar, and continuing distally along the external oblique ridge (Figure 1A). In Group 2, the envelope flap was created with a horizontal linear incision parallel to the free gingival margin, extending from the mesial aspect of the second molar to the retromolar area without vertical releasing incisions (Figure 1B).

Both incisions were made using a no. 15 scalpel blade (Kehr Surgical Pvt. Ltd., Kanpur, India) mounted on a Bard-Parker handle (Hu-Friedy, Chicago, USA). A standard inferior alveolar and lingual nerve block was administered, supplemented by buccal infiltration as needed [8].

Following flap elevation, the impacted mandibular third molar was exposed buccally using a periosteal elevator (GDC Marketing; Hoshiarpur, India). Bone guttering was performed using a no. 8 round bur and straight fissure bur (SS White, Lakewood, USA) under copious irrigation with sterile saline (Aculife Healthcare Pvt. Ltd., Ahmedabad, India) using a 50 mL syringe (Dispovan, Hindustan Syringes & Medical Devices Ltd., Faridabad, India). When necessary, tooth sectioning was performed using a micromotor handpiece (NSK; Nakanishi Inc., Tochigi, Japan) and a straight fissure bur. After extraction, the alveolus was inspected, curetted to remove granulation tissue, and irrigated with sterile saline solution. Primary closure was achieved using 3-0 silk sutures (Ethicon, Johnson & Johnson, New Brunswick, NJ, USA) using a vertical mattress suturing technique. The sutures were removed on postoperative day 7. Postoperatively, all patients received standard antibiotics: amoxicillin 500 mg (Amoxil, GlaxoSmithKline, Brentford, United Kingdom) three times daily for five days and metronidazole 400 mg (Flagyl, Abbott Laboratories, Chicago, USA) three times daily for five days. Analgesics (ibuprofen 400 mg, Brufen, Abbott Laboratories, Chicago, IL, USA) were prescribed as needed.

**Figure 2:**
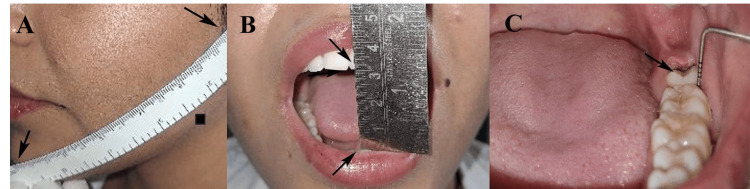
Outcome measures assessed in the study: (A) facial swelling (mm), (B) trismus measured as maximum mouth opening (mm), and (C) probing pocket depth (mm) distal to the mandibular second molar. Original intraoral images of patients from the study were used with the patients’ permission.

The outcome assessment focused on four parameters: pain, trismus or jaw stiffness measured as maximum mouth opening, facial swelling, and probing pocket depth. Pain was evaluated using the visual analog scale (VAS), where patients marked their pain perception on a 10-cm scale (0 = no pain, 10 = severe pain) on postoperative days 1, 3, and 7 [12]. Facial swelling was assessed using the metric method, measuring three facial lines: Gn-Lc line (from the lateral canthus of the eye to the gonion), Tr-Com line (from the tragus of the ear to the commissure of the mouth), and Tr-Pog line (from the tragus of the ear to the pogonion) [13]. These measurements were taken preoperatively and on postoperative days 1, 3, and 7 using a flexible measuring tape (Freemans, Ludhiana, India), and the differences were analyzed to determine the extent and regression of swelling (Figure 2A). Mouth opening was measured as the maximum interincisal distance using a calibrated metallic scale (Camel, Mumbai, India), recorded preoperatively and on postoperative days 1, 3, and 7 [8]. Normal preoperative mouth opening ranged from 35 mm to 55 mm, and postoperative values were compared to assess trismus severity and duration (Figure 2B). The calibration of the metallic scale was verified before each measurement to ensure an accuracy within ±1 mm. The probing pocket depth was measured distally to the second molar using a periodontal probe (Hu-Friedy, Chicago, IL, USA) on the same follow-up days to assess periodontal health (Figure 2C).

Patients were recalled for follow-up on postoperative days 1, 3, and 7 and at 3 months to monitor long-term outcomes. All measurements were performed by a single trained examiner to ensure consistency, with calibration sessions conducted before the study to minimize intra-examiner variability. Data were recorded in a standardized proforma and analyzed to compare the postoperative sequelae between the envelope flap and triangular flap groups with modified Ward’s incision, focusing on differences in pain, trismus, swelling, and periodontal health.

Statistical analysis

Data were analyzed using Statistical Package for Social Sciences (SPSS) software version 20 (IBM Corp., Armonk, NY). The normality of continuous variables, including VAS scores, swelling, trismus, and probing depth, was verified using the Shapiro-Wilk test. Parametric statistical tests were selected for the analysis because the data followed a normal distribution. Independent samples t-tests were employed to compare outcomes between groups, while repeated-measures analysis of variance (ANOVA) was used to assess changes within groups across different time intervals. A p-value threshold of ≤0.05 was established to determine statistical significance for all tests.

## Results

The baseline characteristics of both groups were comparable, with no significant differences in age (p=0.569) or sex (p=0.145). These findings ensured that the groups were balanced for subsequent comparison (Table 1).

**Table 1:** Baseline characteristics of the study groups. p>0.05 indicates no statistically significant difference, as determined by the independent t-test for age and the chi-square test for sex distribution. Age is presented as mean ± standard deviation, and sex distribution is presented as frequency (n) and percentage (%), where n represents the number of patients in each group.

Parameters	Group 1 (triangular flap with the modified Ward’s incision)	Group 2 (envelope flap)	Test statistics	p-value
Age (years)	28.7±6.23	32.8±8.75	1.23	0.569
Male	12 (60%)	10 (50%)	0.34	0.145
Female	8 (40%)	10 (50%)		

Repeated-measures ANOVA revealed significant changes in all parameters in both groups (p<0.001). For VAS scores, both groups showed progressive pain reduction from preoperative levels (triangular flap with modified Ward’s incision: 7.80±0.83; envelope flap: 7.90±0.85) to day 7 (triangular flap with modified Ward’s incision: 1.80±0.83; envelope flap: 1.25±0.91), with the envelope flap group demonstrating greater improvement. Facial swelling peaked at day 1 (triangular flap with modified Ward’s incision: 119.80±5.29 mm; envelope flap: 117.68±6.27 mm) before subsiding by day 7, while mouth opening showed maximum improvement at day 1 (triangular flap with modified Ward’s incision: 22.70±5.27 mm; envelope flap: 21.50±4.47 mm) with gradual recovery thereafter. The probing pocket depth increased postoperatively but improved by 3 months, though the envelope flap group maintained slightly higher values. Notably, the envelope flap technique showed superior pain control (lower VAS) but slightly delayed soft tissue recovery (higher probing depth at 3 months), as shown in Table 2.

**Table 2:** Intragroup (within the group) comparison for parameters using repeated-measures analysis of variance (ANOVA). *p<0.05 indicates statistical significance as determined by repeated-measures ANOVA. Data are presented as mean ± standard deviation. VAS, Visual analog scale

Parameters	Time interval	Group 1 (triangular flap with the modified Ward’s incision)	F value	p-value	Group 2 (envelope flap)	F value	p-value
VAS score	Preoperative	7.80±0.83	2.34	0.001*	7.90±0.85	263.32	0.001*
	Day 1	7.15±1.09			7.00±1.26		
	Day 3	4.75±0.91			4.40±1.47		
	Day 7	1.80±0.83			1.25±0.91		
Facial swelling (mm)	Preoperative	113.45±5.71	60.69	0.001*	112.31±6.74	20.86	0.001*
	Day 1	119.80±5.29			117.68±6.27		
	Day 3	121.33±6.85			116.22±8.52		
	Day 7	116.50±5.67			113.12±7.34		
Maximum mouth opening (mm)	Preoperative	43.15±5.95	106.9	0.001*	41.60±5.71	64.62	0.001*
	Day 1	22.70±5.27			21.50±4.47		
	Day 3	23.70±4.09			23.85±7.95		
	Day 7	32.50±5.73			33.50±6.24		
Probing pocket depth (mm)	Preoperative	2.12±0.44	39.27	0.001*	2.20±0.60	139.13	0.001*
	Day 7	3.03±0.56			4.58±0.90		
	3 months	1.90±0.34			2.22±0.38		

Intergroup analysis revealed a significant increase in probing pocket depth from preoperative to day 7 (mean difference = 1.47 mm, p<0.001), indicating immediate postoperative tissue changes. However, by 3 months, the difference became non-significant (mean difference = 0.21 mm, p=0.157), demonstrating comparable long-term healing between groups (Table 3).

**Table 3:** Comparative analysis between the groups for mean change in probing depth using the independent t-test at multiple time intervals. *p<0.05 indicates statistical significance as determined by the independent t-test. The mean difference was calculated by subtracting the mean change in probing depth of the triangular flap group from that of the envelope flap group.

Parameter	Mean change in probing depth from preoperative to day 7			Mean change in probing depth from preoperative to 3 months		
	Mean difference (mm)	t value	p-value	Mean difference (mm)	t value	p-value
Probing depth (mm)	1.47	6.38	0.001*	0.21	1.44	0.157

Comparative analysis revealed significant differences in facial swelling scores between groups at days 3 and 7, with Group 2 showing better improvement in facial swelling. The improvement in mouth opening was significant only on day 3, suggesting transient differences in maximum mouth opening recovery, which was better in Group 2. The improvement in pain scores showed no significant intergroup differences at any time point, indicating comparable relief of postoperative pain. The most pronounced between-group variation occurred in facial swelling, particularly during the subacute phase (day 3), while early (day 1) and late (day 7) assessments demonstrated modest differences. These findings highlight that while both techniques manage pain equally well, improvement in facial swelling varies significantly during the critical healing phases, with Group 2 maintaining superior effects (Table 4).

**Table 4:** Comparative analysis of clinical parameters between groups using the independent t-test at multiple time intervals. *p<0.05 indicates statistical significance using the independent t-test. The mean difference was calculated by subtracting the mean change in clinical parameters of the triangular flap group from that of the envelope flap group. VAS, Visual analog scale

Time intervals	Facial swelling (mm)			VAS scores			Mouth opening (mm)		
	Mean difference	t value	p-value	Mean difference	t value	p-value	Mean difference	t value	p-value
Mean change from preoperative to day 1	0.98	1.24	0.222	0.25	1.39	0.174	0.35	0.25	0.801
Mean change from preoperative to day 3	3.99	3.32	0.002*	0.45	1.32	0.194	1.70	1.63	0.031*
Mean change from preoperative to day 7	2.26	2.78	0.008*	0.65	1.75	0.089	2.55	1.13	0.267

## Discussion

The findings of this prospective study highlight the envelope flap as a preferable surgical approach over the triangular flap with a modified Ward’s incision for lower third molar disimpaction, particularly in reducing postoperative facial swelling and facilitating earlier recovery of mouth opening. This superiority stems from the envelope flap design, which relies on a sulcular incision that avoids vertically releasing cuts, thereby limiting soft tissue disruption, vascular compromise, and subsequent inflammatory cascades. In contrast, the triangular flap's anterior vertical incision extends from the distal aspect of the first molar, potentially increasing hematoma formation and edema by exposing more bone and periosteum, exacerbating tissue trauma [8]. Such mechanistic differences align with the observed variations in the postoperative sequelae, underscoring the role of the envelope flap in enhancing patient comfort during the acute healing phase.

The advantage of the envelope flap in alleviating facial swelling, especially during the subacute period, may be explained by its preservation of mucoperiosteal integrity, which promotes better lymphatic drainage and reduces interstitial fluid accumulation. This is consistent with biomechanical principles, where minimal incision length correlates with decreased cytokine release and inflammatory mediator activation, as observed in surgical models emphasizing tissue-sparing techniques [14]. Serum IL-8, which has been observed to be markedly increased in rats subjected to long incisions compared to those subjected to short incisions, serves as a neutrophil chemoattractant peptide that is released during the initial phase of the immune response to facilitate the recruitment of neutrophils to the site of inflammation [15]. The triangular flap with a modified Ward’s incision has a longer incision length than the envelope flap due to the additional vertical anterior incision. This extended incision facilitates greater flap mobility and exposure but contributes to the increased postoperative swelling and trismus noted in this study.

A meta-analysis by Zhu et al. [16] corroborated this by noting that envelope flaps are associated with overall reduced postoperative pain and trismus in class A and B impactions. However, their review did not isolate swelling. This could arise from our focused measurement of facial dimensions (Gn-Lc, Tr-Com, and Tr-Pog lines) that captured subtle volumetric changes not always quantified in broader syntheses. Additionally, the increased facial swelling observed in the triangular flap group may be partly attributed to the longer surgical time associated with this technique. The triangular flap design, incorporating a vertical anterior incision, requires extensive dissection and flap reflection compared with the simpler sulcular incision of the envelope flap. Prolonged operative duration can exacerbate tissue trauma, leading to heightened release of biological mediators, such as prostaglandins, histamine, and cytokines, which amplify the inflammatory response and contribute to postoperative edema, pain, and trismus. This is supported by Bailey et al. [17], who noted that extended surgical manipulation in third molar extractions is correlated with increased inflammatory sequelae owing to sustained tissue exposure and stress. The streamlined approach of the envelope flap likely minimizes operative time, reduces the inflammatory cascade, and aligns with its observed superiority in controlling swelling during the subacute healing phase.

Furthermore, the transient improvement in mouth opening observed with the envelope flap likely reflects its indirect mitigation of trismus through reduced edema impacting masticatory muscles, such as the masseter and medial pterygoid. Trismus arises from reflexive muscle spasm secondary to inflammation, and reduced dissection of the envelope flap minimizes this reflex, as supported by Lopes da Silva et al. [18]. They found no overarching differences in trismus but highlighted flap-specific influences on early mobility in their systematic review. However, the authors noted greater postoperative ecchymosis with the triangular flap design.

Regarding pain management, the envelope flap's trend toward lower intensity can be attributed to decreased nociceptor stimulation due to limited flap elevation and bone exposure [14,15]. The absence of a vertical incision reduces tension on the wound margins during closure, potentially lowering mechanical irritation and neuropathic signals from the branches of the inferior alveolar nerve. This interpretation is bolstered by Zhu et al. [16], whose meta-analysis demonstrated significantly lower VAS scores on days 3 and 7 with envelope flaps in similar impaction subtypes. They attribute this to shorter operative times and less aggressive manipulation, factors which are not directly measured here but inferred from procedural standardization. Although pain relief was equivalent between groups in terms of overall trajectory, the envelope flap's edge suggests that it better accommodates patients with lower pain thresholds, possibly by preserving a more intact gingival architecture. In contrast, Bailey et al. [17] and Zhu et al. [16] noted a lower incidence of alveolar osteitis with a triangular flap than with the envelope flap design.

The comparable long-term periodontal outcomes, despite a marginal elevation in probing depth with the envelope flap, indicate that both techniques support eventual tissue remodeling without compromising distal second molar health. Higher initial depths in the envelope group may result from the proximity of the sulcular incision to the periodontal ligament, fostering temporary pocket formation due to delayed reattachment. However, a resolution of 3 months implies robust gingival healing driven by fibroblast proliferation and collagen maturation. This finding was supported by Lopes da Silva et al. [18], who reported lower probing depths on day 7 with triangular flaps, suggesting early advantages in plaque control and epithelial sealing. However, our non-significant long-term differences emphasize that envelope flaps do not pose enduring risks, particularly in systemically healthy patients. Hur and Ogata [19] conducted a systematic review to assess the effects of different flap designs on the periodontal conditions surrounding the mandibular second molars following third molar extraction, and they concluded that the choice of flap design does not affect the periodontal health of the second molars. Consequently, the selection of the flap design is related to personal preference.

These interpretations are grounded in the interplay between surgical anatomy and wound healing physiology, where flap design influences hemostasis, infection risk, and scar formation. The horizontal orientation of the envelope flap aligns with Langer's lines, minimizing tensile stress and promoting cosmetic outcomes, whereas the triangular flap configuration provides superior visualization for complex angulations. The evaluations conducted 3 months after extraction indicated optimal healing in relation to both flaps. Most previous studies have conducted evaluations up to seven days postoperatively [7,8,20]. Follow-up was performed for 3 months in our study, which is considered sufficient. Extending this timeframe may result in a higher likelihood of patient attrition during follow-up.

The clinical implications of these results suggest that the envelope flap can be a default choice for uncomplicated mandibular third molar extractions, especially for patients prioritizing rapid aesthetic and functional recovery, such as young adults or those in public-facing roles. It may reduce reliance on anti-inflammatory adjuncts and improve compliance and cost-effectiveness. Surgeons should weigh case-specific factors, such as impaction depth, and reserve triangular flaps for scenarios that demand extended access to mitigate potential alveolar osteitis, as noted in prior reviews [16,17].

Limitations include non-randomized allocation, which may introduce selection bias based on the surgeon's preference and tooth angulation, potentially confounding the outcomes. The modest sample size limits generalizability, particularly to Position C impactions or diverse ethnic populations, and the single-center design overlooks inter-operator variability. Future randomized trials with larger cohorts and blinded evaluators are needed to validate these findings.

## Conclusions

This prospective study demonstrated that the envelope flap is superior to the triangular flap with a modified Ward’s incision for lower third molar disimpaction, primarily because of its significant reduction in postoperative facial swelling and earlier improvement in mouth opening during the subacute healing phase. Both flap designs achieved comparable pain control and long-term periodontal health, with primary closure ensuring effective healing by primary intention. These findings advocate the preferential use of the envelope flap in uncomplicated mandibular third molar extractions, especially for patients who prioritize rapid functional and aesthetic recovery. However, the triangular flap remains valuable in cases that require enhanced surgical access. Future randomized studies with larger cohorts and extended follow-up are recommended to validate these findings further and to explore additional factors that may influence postoperative outcomes, such as operative duration.
